# Genomic and phenotypic characterisation of *Pseudomonas aeruginosa* isolates from canine otitis externa reveals high-risk sequence types identical to those found in human nosocomial infections

**DOI:** 10.3389/fmicb.2025.1526843

**Published:** 2025-02-24

**Authors:** Bailey Secker, Stephen Shaw, Laura Hobley, Robert J. Atterbury

**Affiliations:** ^1^School of Biosciences, University of Nottingham, Nottingham, United Kingdom; ^2^School of Veterinary Medicine and Science, University of Nottingham, Nottingham, United Kingdom

**Keywords:** pseudomonas, canine, otitis externa, genome, AMR, biofilm, one health, nosocomial

## Abstract

**Introduction:**

Canine otitis externa (OE) is a frequently-diagnosed condition in veterinary practices worldwide. *Pseudomonas aeruginosa* is commonly associated with chronic and recalcitrant canine OE, but studies with detailed genomic and phenotypic characterisation of clinical isolates are lacking.

**Methods:**

*Pseudomonas aeruginosa* canine OE isolates (*n* = 253) were collected from different geographical locations in Europe and characterised with respect to antimicrobial resistance and biofilm formation. A subset (*n* = 35) were genome sequenced then characterised with respect to diversity, and complement of virulence, antimicrobial resistance, and biofilm-associated genes.

**Results:**

Genome-sequenced *P. aeruginosa* strains were distributed among phylogroups, showing no obvious clonality. However, two isolates belonged to ST111 and ST244 respectively,—MLST sequence types associated with AMR nosocomial infections in humans. Resistance to fluoroquinolones was detected in 25% of isolates, and multidrug resistance detected in 1.6%, though this did not always correlate with the presence of antimicrobial resistance genes. Additionally, 82% of isolates were characterised as forming strong biofilms.

**Discussion:**

For the first time, this study has characterised a large multinational collection of *P. aeruginosa* isolates from canine otitis with a combination of whole genome sequencing, phenotypic screening and bioinformatic analysis. These strains did not cluster together based on genomic diversity or virulence gene complement, supporting their likely environmental origin. However, the identification of ST111 and ST244, important ‘high-risk’ sequence types, could suggest potential spread between humans and dogs. Furthermore, we found that most strains were formed strong biofilms, and exhibited a significant level of resistance towards critically important antimicrobials. These findings could assist in the selection of appropriate treatments for canine OE as well as possibly identifying one health risks of these infections for cohabiting pets and humans.

## Introduction

Canine otitis externa (OE) is recognised as inflammation of the ear canal and is one of the most common diagnoses affecting between 7.3–10.2% of dogs attending UK primary care practices (O’Neill et al., 2014; O’Neill et al., 2021). A similar prevalence ranging from 6.3–13%, has been reported worldwide (Lund et al., 1999; Kim et al., 2018). OE is thought to result from secondary infection following a multifactorial primary inflammation (Paterson and Matyskiewicz, 2018). *Pseudomonas aeruginosa* is the most common pathogen isolated from chronic and recalcitrant canine OE; associated with up to 35% of cases (Nuttall and Cole, 2007). *Pseudomonas aeruginosa* is a Gram-negative, opportunistic pathogen of humans and animals. In humans, it is closely associated with cystic fibrosis patients, where in the UK 13.1% of adults suffer from chronic *P. aeruginosa* infections and 18.8% from intermittent infections (Naito et al., 2023). A similar prevalence can be seen globally in the US, Canada and Germany (Cystic Fibrosis Canada, 2023; Cystic Fibrosis Foundation, 2023; Nährlich et al., 2023).

Treatment of *P. aeruginosa* canine OE usually requires the use of antimicrobials as part of combined steroid, antimicrobial, antifungal products. Three classes of antimicrobials are often used; fluoroquinolones such as marbofloxacin; aminoglycosides (particularly gentamicin); and polymyxin B (Secker et al., 2023). Treatment is challenging because of tissue swelling, hyperplasia and antimicrobial resistance (AMR). *Pseudomonas aeruginosa* has intrinsic AMR mechanisms such as limited outer membrane permeability, efflux pumps and a chromosomally encoded β-lactamase (Cox and Wright, 2013). It can also rapidly accrue mutations conferring resistance to most antimicrobials used in clinical therapy for OE (López-Causapé et al., 2018) or acquire antimicrobial resistance genes (ARG) through horizontal gene transfer (Pang et al., 2019).

The majority (>90%) of *P. aeruginosa* isolates from clinical cases of OE in both humans and dogs have been shown to produce biofilms (Fusconi et al., 2011; Robinson et al., 2019). This reduces treatment success as *P. aeruginosa* cells within a biofilm can be many times more resistant to antimicrobials than planktonic cells (Thöming and Häussler, 2022). Consequently, this can result in incurable chronic infections which—in extreme cases—require surgery (Pye, 2018).

Whole genome sequencing has not been used to evaluate *P. aeruginosa* isolates from clinical cases of canine OE to our knowledge. As such, little is known about the full genetic diversity of these isolates. Likewise, few studies have investigated the presence and role of virulence genes in *P. aeruginosa* isolates from canine OE. One study found a high prevalence of five virulence genes from canine *P. aeruginosa* isolates, though these were not exclusively from OE infections (Hattab et al., 2021). Multilocus Sequence Typing (MLST) studies have found a range of sequence types (ST) linked to *P. aeruginosa* from canine otitis and pyoderma which hinted at the underlying genetic diversity amongst these isolates (Hyun et al., 2018; Elfadadny et al., 2023).

In the present study, antimicrobial resistance and biofilm formation was assessed for 253 European isolates of *P. aeruginosa* from clinical cases of canine OE. Subsequently, a subset of 35 isolates representing different phenotypes were selected for whole genome sequencing to determine their complement of virulence and antimicrobial resistance genes (ARG) and to gain a fuller understanding of their genomic diversity.

## Materials and methods

### Bacterial strain isolation and laboratory maintenance

A collection (*n* = 253) of geographically diverse *Pseudomonas aeruginosa* isolates from dogs with otitis externa were collected from the UK Royal Veterinary College (RVC; *n* = 48), CAPL Nationwide Laboratories, UK (*n* = 99), and an EU collection from the University of Copenhagen (*n* = 106). Confirmation of *P. aeruginosa* identity was performed as described by the UK Health Security Agency standards for microbiology investigations (UKHSA, 2024) using cetrimide agar and the oxidase test, followed by 16S PCR. *Pseudomonas aeruginosa* PAO1 was used as a positive control for all assays in this study.

Prior to each experiment, agar plates were prepared [Lysogeny Broth (LB), Miller, 25 g/L, Sigma-Aldrich; supplemented with agar, 15 g/L, Fisher Scientific], and were inoculated with the required *P. aeruginosa* strain and streaked to obtain single colonies. Plates were inverted and incubated statically at 37° C for 16 h. Following incubation, 10 mL of LB was inoculated with a single colony using a sterile inoculating loop and incubated at 37° C with shaking at 200 rpm for 16 h.

### Antimicrobial sensitivity testing

Antimicrobial sensitivity testing (AST) was performed as described by the Clinical & Laboratory Standards Institute (CLSI) using the disk diffusion method (CLSI, 2012). A suspension of bacterial cells was prepared by inoculating 8 mL of Mueller-Hinton broth (MH; Sigma-Aldrich) with bacterial colonies selected from an LB plate until a turbidity equivalent to a 0.5 McFarland standard had been reached, as determined by visual comparison using a Wickerham card. A swab was then used to inoculate the surface of a MH agar 2 plate (Sigma-Aldrich) ensuring the entire plate is covered evenly by rotating the plate 90° between swabbing the first time with a final diagonal swab. The plates were then incubated at 37°C for 16 h, and the results recorded by measuring the inhibition zone diameter using a digital calliper.

AST was performed using an antimicrobial panel comprising six antimicrobial classes (Supplementary Table 1). All antimicrobials were tested against all strains except for Ticarcillin + Clavulanic acid which was discontinued part way through this study, and were acquired from two suppliers: ProLabs and Oxoid. *E. coli* ATCC 25922 and *P. aeruginosa* ATCC 27853 were the control strains used in this assay. Antimicrobial sensitivity was determined using breakpoints outlined by the CLSI, where available canine veterinary breakpoints were used (CLSI, 2018b) otherwise human breakpoints were used (CLSI, 2018a).

### Submerged biofilm assay

The ability of clinical *P. aeruginosa* isolates to form biofilm was tested *in vitro* as described previously with some modifications (Coffey and Anderson, 2014). Briefly, overnight cultures were diluted 1 in 100 into LB and 100 μL aliquots were separately transferred to wells of the 96-well microtiter plates in triplicate in the same microtiter plate; two microtiter plates were used for each technical repeat. A negative control, comprising three wells containing sterile LB, was included on each plate. The cultures were then grown statically at 37° C for 24 h. Following incubation, planktonic cells were removed, and the wells were washed using 125 μL of Ca/HEPES buffer; the wells were then stained using 0.1% (v/v) crystal violet solution. Excess crystal violet was removed, and the wells were then de-stained using ethanol (100%), and absorbance measured at 595 nm using a Tecan GENios Pro. The assay was repeated to achieve three biological repeats. Biofilm production was scored as either as either strong, moderate, weak, or non-biofilm producing as previously described (Stepanović et al., 2000).

### Swarming motility assay

Swarming motility assays were modified according to (Ugurlu et al., 2016). Briefly, 1 μL of bacterial culture was spotted into the centre of swarming motility agar (8 g/L nutrient broth, Oxoid; 5 g/L agar select, Sigma-Aldrich; 0.5% v/v glucose, Fisher Scientific), and then incubated at 37° C for 16 h. The distance travelled from the point of inoculation was measured using a digital calliper, and swarming motility assessed by phenotype on the plates in combination with the distance travelled from the point of inoculation.

### *Pseudomonas aeruginosa* genome sequencing

Whole genome sequencing of 35 isolates was performed using both short (Illumina MiSeq) and long (Oxford Nanopore) read technologies by MicrobesNG (Birmingham, United Kingdom). Isolates were selected for sequencing to ensure isolates from the CALP nationwide laboratories (*n* = 13), Denmark (*n* = 7) and the RVC (*n* = 9) were represented (PRJNA1180571) and included some sequences from previous work (*n* = 6; Gigante et al., 2024; PRJNA107813).

Illumina paired-end reads were quality-assessed and trimmed using FastQC v0.11.8 (Andrews, 2010) and FastP v0.12.4 (Chen et al., 2018) respectively. Subsequently, contigs were assembled *de novo* using Flye v2.9.2-b1786 (Kolmogorov et al., 2019), followed by post-processing with Circlator v.1.5.5 (Hunt et al., 2015) and Bandage v0.8.1 (Wick et al., 2015). This was followed by one round of long read polishing and two rounds of short read polishing using Medaka v1.11.1 (ONT, 2023), Polypolish v0.5.0 (Wick and Holt, 2022) and POLCA from the MaSuRCA toolkit v4.0.9 (Zimin and Salzberg, 2020) respectively. Finally, genomes were reoriented to begin with *dnaA* using dnaapler v0.4.0 (Bouras et al., 2024) and annotated using Bakta v1.8.2 (Schwengers et al., 2021).

### Bioinformatic analysis

To aid in the comparison of isolates from the present study to a wider population of *P. aeruginosa* isolates, PanACoTA v1.4.0 (Perrin and Rocha, 2021) was used to download and filter *P. aeruginosa* genomes from the RefSeq database as previously described (Botelho et al., 2023). Briefly, *P. aeruginosa* sequences were selected using taxid (−T 287) and filtered so that genomes with more than 100 contigs were removed (--nbcont 100). Subsequently, redundant and misclassified genomes were removed using Mash distances (--min_dist 0.0001; --max_dist 0.05). Metadata for the RefSeq genomes was downloaded using NCBI Datasets v16.40.1 (O’Leary et al., 2024).

Whole genome average nucleotide identity (ANI) was calculated using FastANI v1.34 (Jain et al., 2018). This was used in combination with hierarchical clustering using Euclidean distance and complete linkage to assign isolates to a phylogroup (Botelho et al., 2023; Supplementary Figure 1). A neighbour joining phylogenetic tree was constructed using Mashtree v1.4.6 (Katz et al., 2019) using bootstrapping with 100 replicates and mindepth 0. Trees were then visualised using iTOL (Letunic and Bork, 2024).

Multilocus sequence typing (MLST) was performed *in silico* using the PubMLST database (Jolley et al., 2018). Where an unknown ST was identified, genome sequences were uploaded to the PubMLST database, and a new ST was assigned.

Screening of virulence genes was performed using ABRicate (Seemann, 2015) using the VFDB (Liu et al., 2021). Acquired antimicrobial resistance genes were identified using AMRFinderPlus v4.0.3 using assembled nucleotide sequences (−n), this included searching for point mutations (−O; Feldgarden et al., 2021).

Biofilm associated genes were identified using a custom database, created as described by (Seemann, 2015). Genes located within the *psl*, *pel* and alginate (*alg*) operons (Franklin et al., 2011) in addition to other associated biofilm genes (Liu et al., 2021; Kiel et al., 2022) were downloaded from NCBI using *P. aeruginosa* PAO1 (NC_002516.2). A complete list of the genes is shown in Supplementary Table 2.

## Results

### Genomic diversity of *Pseudomonas aeruginosa* from canine otitis infections

**Figure 1:**
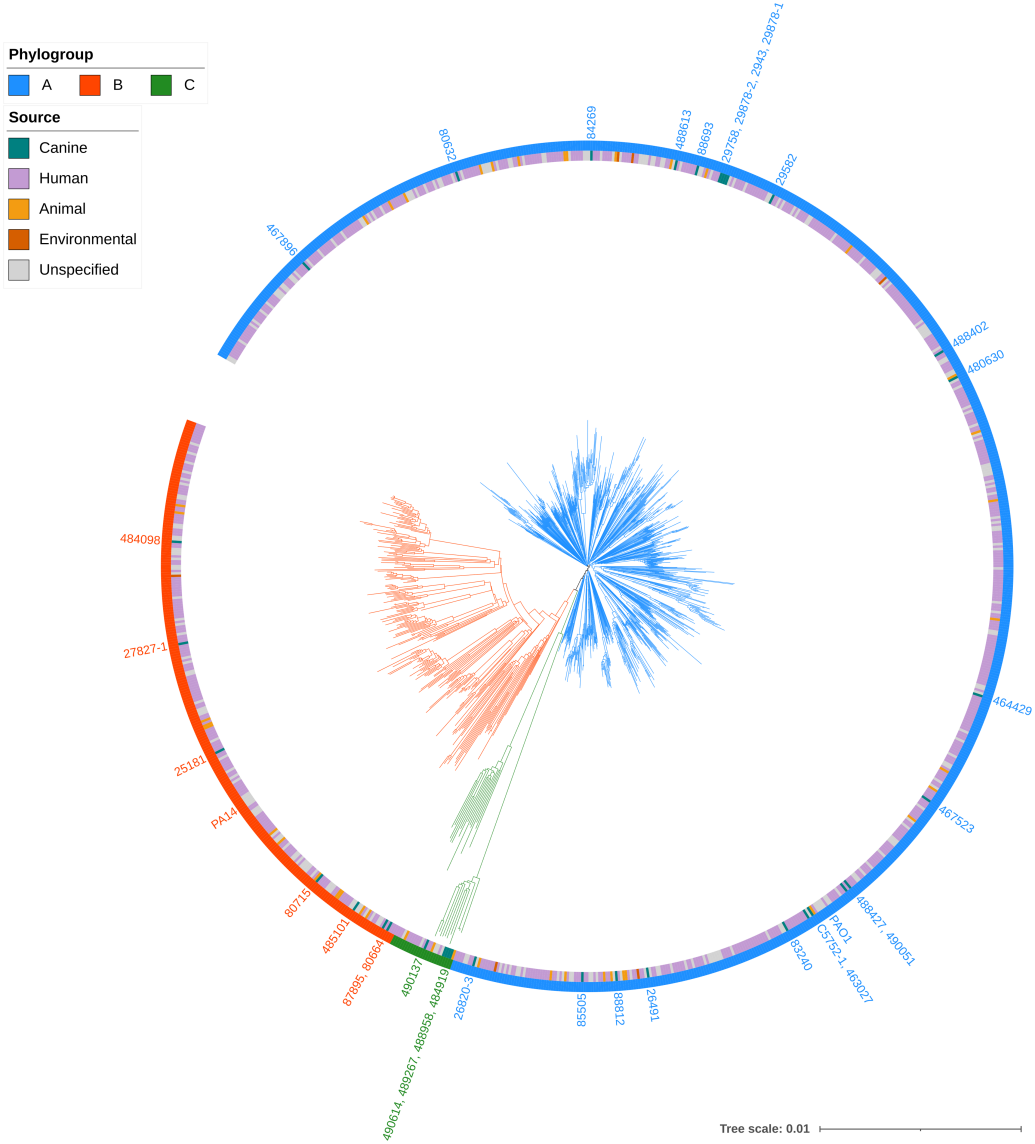
Phylogenetic tree of *Pseudomonas aeruginosa* strains. Mashtree with bootstrapping (100 replicates) was used to calculate the phylogenetic distances of the *P. aeruginosa* isolates from this study in addition to 1,000 isolates from other sources to create a neighbour joining tree which was visualised using iTOL. The scale represents 1 base difference per 100 bases. The outer ring represents the phylogroups while the inner ring shows the source of the isolates (where available). Strains from the present study, in addition to PAO1 and PA14, are labelled on the tree, coloured according to their phylogroup.

A total of 10,300 genomes were downloaded from the RefSeq database and filtered for quality and genetic distance. The resulting set of 3,721 genomes was then randomly subsampled to yield a subset of 1,000 genomes. This included *P. aeruginosa* PAO1 (GCF_000006765.1) and PA14 (GCF_000014625.1) but PA7 (GCF_000017205.1) was removed based on Mash genetic distance which is in line with previous reports proposing that this strain be reclassified to a novel species *Pseudomonas paraeruginosa* (Rudra et al., 2022). These genomes in addition to 35 from the present study were compared using Mashtree. The distances calculated were then used to construct a phylogenetic tree (Figure 1). The strains were ascribed to three phylogroups; A (*n* = 765), containing *P. aeruginosa* PAO1; B (*n* = 244), containing *P. aeruginosa* PA14, and a third minor group, C (*n* = 26). The classification of *P. aeruginosa* into three phylogroups has been described previously (Stewart et al., 2014; Ozer et al., 2019); with one study suggesting that phylogroup C should be split into three groups (Freschi et al., 2019). However, with the exclusion of PA7, the results in the present study are in line with a recent study of *P. aeruginosa* phylogeny (Botelho et al., 2023).

Canine strains from the present study were distributed across all three phylogroups indicating that they were not closely related and did not cluster according to host source or geographical origin. This was supported by MLST analysis which identified 30 different STs among the clinical isolates. One isolate, 488613, was assigned a novel ST (5180). The most common ST was 788 which was identified in 9% (3/35) of clinical isolates.

### Antimicrobial sensitivity testing revealed resistance to up to six antimicrobials

**Figure 2:**
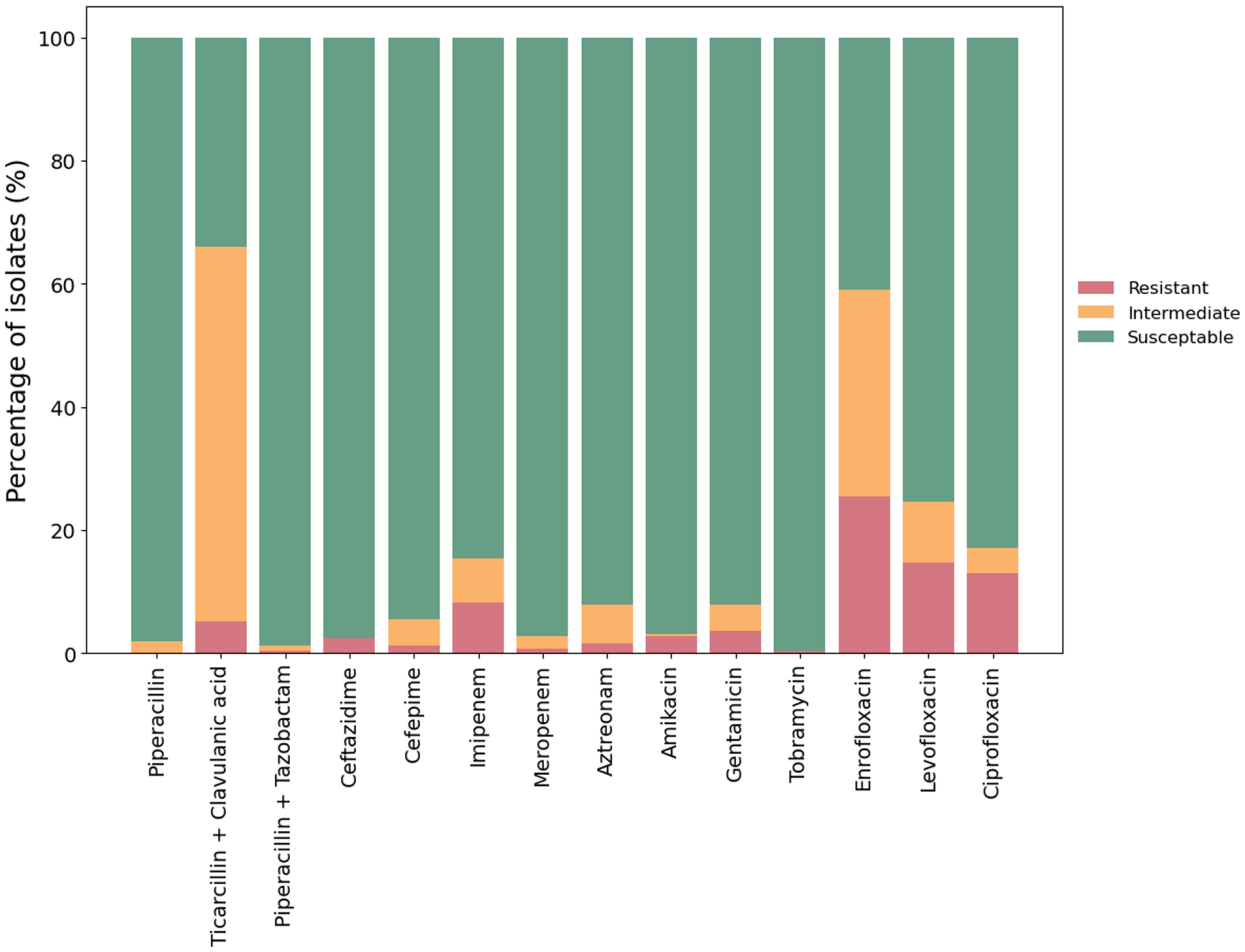
Antimicrobial susceptibility of *P. aeruginosa* isolates. The antimicrobial susceptibility of 252 *P. aeruginosa* isolates from clinical cases of canine otitis externa was determined using the disk diffusion method (CLSI, 2012). The percentage of sensitive, intermediate and resistant isolates are shown with red, amber and green bars, respectively.

Antimicrobial resistance (AMR) in *P. aeruginosa* from cases of canine otitis externa was determined using the disk diffusion method with 14 antimicrobials from 6 antimicrobial classes (Figure 2). Isolate 29130 failed to grow under the tested conditions and was excluded from this assay. The highest level of resistance was recorded for enrofloxacin (25.4%; 64/252) while tobramycin showed the highest sensitivity (99.6%; 251/252). Intermediate resistance was mainly seen against ticarcillin + clavulanic acid (60.7%; 127/252). Finally, 8.3% (21/252) and 0.79% (2/252) of isolates were resistant to imipenem and meropenem, respectively.

**Figure 3:**
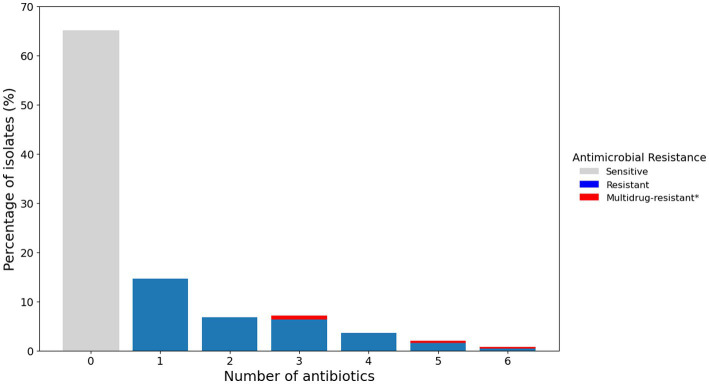
Percentage of *P. aeruginosa* isolates from canine otitis externa resistant to multiple antimicrobials. Bar chart representing the percentage of isolates resistant to a different number of antimicrobials as determined by the disk diffusion method. *Multidrug resistance is reported for resistance to three or more classes of antimicrobials.

Of the 252 isolates tested, 65% (164/252) were susceptible to all of the tested antimicrobials. The remaining 35% (88/252) were resistant to at least one antimicrobial. Moreover, 1.6% (4/252) were classified as multidrug resistant—defined as resistant to three or more different classes of antimicrobials (Sweeney et al., 2018; Figure 3).

### Comparison of genotypic and phenotypic antimicrobial resistance

**Figure 4:**
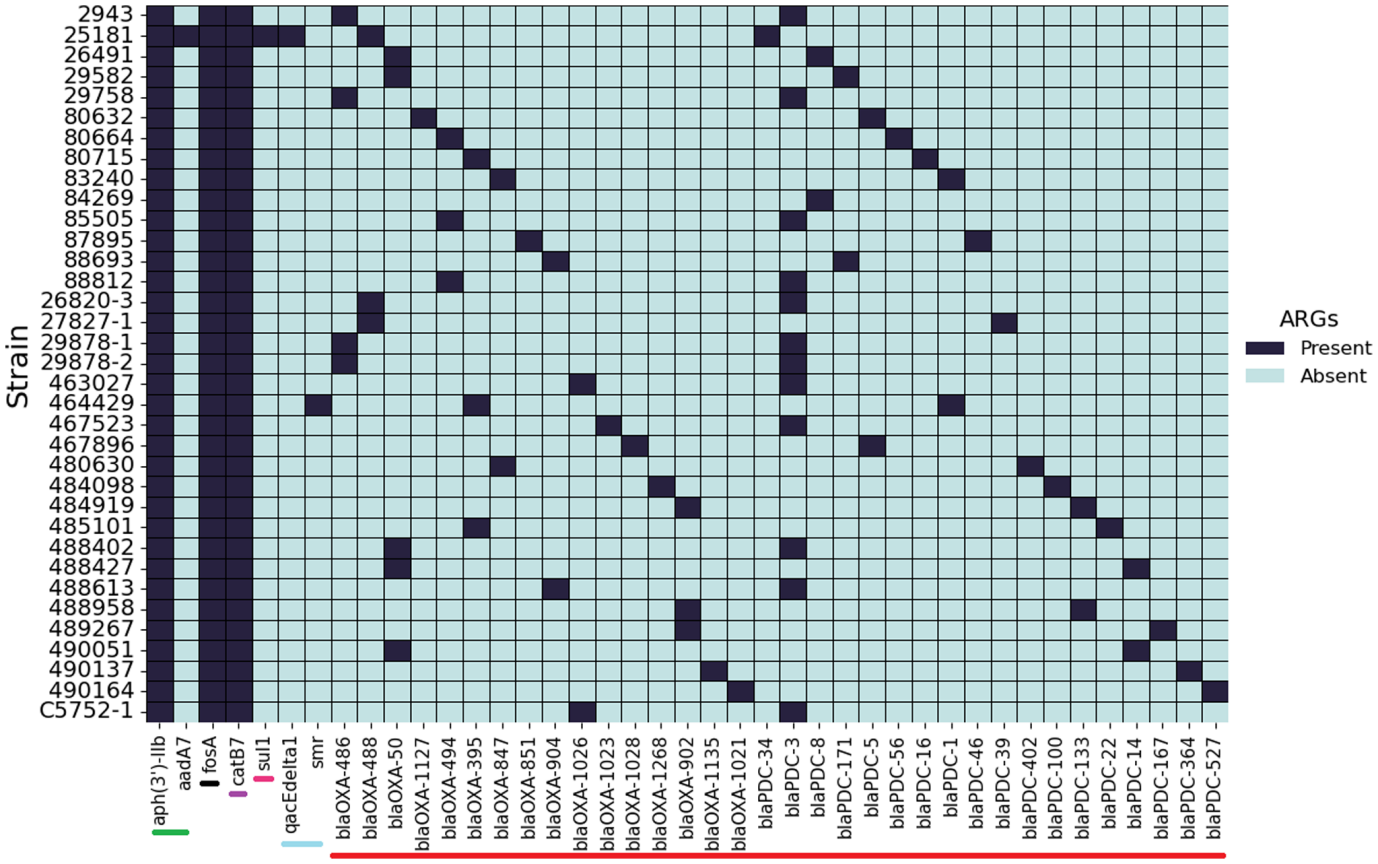
Presence and absence of antimicrobial resistance genes. Screening of 35 *P. aeruginosa* isolates from canine otitis infections for the presence of antimicrobial resistance genes. Bars at the base of the figure indicate the antibiotic class: Green—aminoglycoside, Black—Fosfomycin, Purple—chloramphenicol, Pink—sulfonamide, Light blue—Quaternary ammonium, Red—β-lactam.

The 35 genome sequences of clinical *P. aeruginosa* isolates from canine OE were screened for the presence of ARG (Figure 4). Despite the variation in phenotypic susceptibility to antimicrobials between the strains, their complement of antimicrobial resistance genes remained remarkably consistent. Five genes, *aph(3′)-IIb, fosA*, *catB7*, *blaOXA* and *blaPDC* were detected in almost all of the isolates. Some notable exceptions are the presence of *sul1*, *aadA7* and *qacEΔ1* in 25181 and the absence of a *blaOXA* gene in 84269. The genes *aadA7* and *sul1* are linked to integrons and encode for a aminoglycoside nucleotidyltransferase that confers resistance to streptomycin and spectinomycin (Ahmed et al., 2004) and a sulfonamide resistant dihydropteroate synthase, respectively, (Sköld, 2000).

Development of resistance through the acquisition of mutations is one of the main drivers of resistance in *P. aeruginosa* (López-Causapé et al., 2018). Twelve different mutations were identified in the present study (Supplementary Table 3); the most common occurring in *oprD*. Other common mutations included those in the quinolone resistance-determining region (QRDR), which are the primary way in which *P. aeruginosa* can become resistant to fluoroquinolones (Piddock, 1999). In *E. coli* these mutations occur between amino acid residues 67–107 for GyrA, and 63–102 for ParC (Egorov et al., 2018). Studies with *P. aeruginosa* have reported the QRDR regions in GyrB as 429–585 and ParE as 357–503 (Bruchmann et al., 2013).

QRDR mutations were identified in almost all of the isolates that were resistant to at least one fluoroquinolone antimicrobial—with the exception of 84269 that was resistant to enrofloxacin but did not contain a QRDR mutation. When examining ARG individually, *gyrA* mutations were the most common, occurring in 20% (7/35) of isolates. The mutation T83I in GyrA was the most common among strains resistant to at least one fluoroquinolone. S466F, S87L/S331T and D533E were the most common mutations in GyrB, ParC and ParE respectively. These are within the QRDR regions, and have been previously associated with resistance (Akasaka et al., 2001; Bruchmann et al., 2013). Furthermore, some of the isolates contained more than one QRDR mutation which is known to further increase the MIC of ciprofloxacin (Bruchmann et al., 2013).

Two of the sequenced strains were resistant to gentamycin. Resistance to aminoglycosides can be due to inactivation by enzymes but also though reduced permeability or increased efflux (Poole, 2005). More recently, mutations to *fusA1* in *P. aeruginosa* have been shown to confer resistance to aminoglycosides (Bolard et al., 2018). One chromosomal aminoglycoside resistance gene, 3′-phosphotransferase (*aph(3′)-IIb*), was identified which conferred resistance to kanamycin A and B, neomycin B and C, butirosin and seldomycin F5 in all the sequenced isolates (Zeng and Jin, 2003). However, *aph(3′)-IIb* does not provide resistance to the clinically relevant aminoglycosides tested in the present study (Atassi et al., 2023).

Only a small number of sequenced isolates (2/35) harboured a mutation in *fusA1*. Strain 488958, which was resistant to all of the tested aminoglycosides, contained T671A. Mutations in domains II, IV and V have been reported to increase the MIC of aminoglycosides up to 16-fold. T671A has been shown to significantly decrease the susceptibility of *P. aeruginosa* to aminoglycosides including gentamicin, amikacin and tobramycin (Bolard et al., 2018). Conversely, strain 87895 contained a Q678L mutation in the *fusA1* gene which is known to result in a four-fold increase in the minimum inhibitory concentration (MIC) of tobramycin, however this strain was sensitive to tobramycin, and other tested aminoglycosides, in the present study (Scribner et al., 2020).

Other mutations identified in the present study include the introduction of a premature stop codon in *mexZ* in 8.6% (3/35) of the isolates. Inactivation of *mexZ* results in the overproduction of the MexXY-OprM efflux pump which can result in decreased susceptibility to fluoroquinolones, aminoglycosides and cefepime (López-Causapé et al., 2018). Finally, 14% (5/35) of the isolates harboured a V15I mutation in the *pmrB* gene, previously identified in clinical strains with an increased MIC of colistin (Lee and Ko, 2014) although this antibiotic was not included in the present study.

### Biofilm-forming ability of *Pseudomonas aeruginosa* isolates

**Figure 5:**
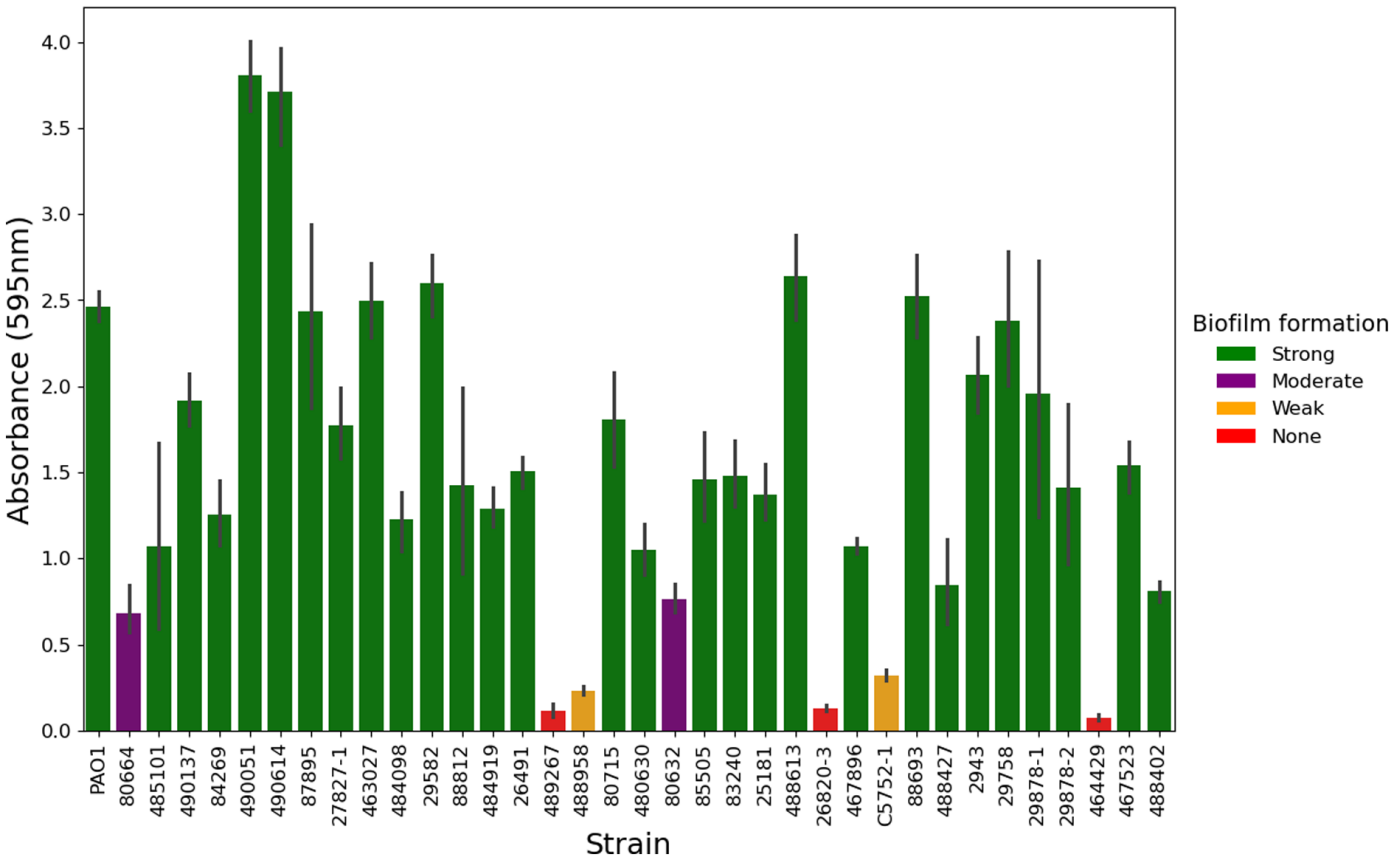
Biofilm formation of *P. aeruginosa* from canine otitis externa. The biofilm forming ability of 253 *P. aeruginosa* strains was tested in a crystal violet, 96 well plate assay, a representative subset of 35 strains is shown here. Green—Strong biofilm producing, Purple—Moderate biofilm producing, Amber—Weak biofilm producing, Red—No quantifiable biofilm. The values here are from three biological and six technical repeats represented by the mean with 95% confidence interval.

Clinical isolates of *P. aeruginosa* were tested for their ability to form a biofilm *in vitro* (Figure 5; Supplementary Figure 2). In total, 82% (207/253) of strains produced strong levels of biofilm, 9% (23/253) produced moderate biofilm, 5% (13/253) produced weak biofilm and 4% (10/253) made no quantifiable biofilm under the tested conditions.

### Detection of biofilm associated genes

To further understand the differences between levels of biofilm formation, the genomes of 35 *P. aeruginosa* isolates were screened for the presence of 53 genes known to be associated with biofilm formation (Supplementary Figure 3). All of the genes were highly conserved when compared to PAO1, with the exception of *algP*—a histone-like regulatory protein whose function in alginate synthesis is disputed and may be strain-dependent (Cross et al., 2020). All but one of the isolates contained all 53 genes, with the exception of 464429 which did not contain *pslABCD*. These genes encode the psl polysaccharide, which contributes to submerged biofilm (Friedman and Kolter, 2004), which may explain the poor biofilm forming ability of this strain.

### Swarming motility

**Figure 6:**
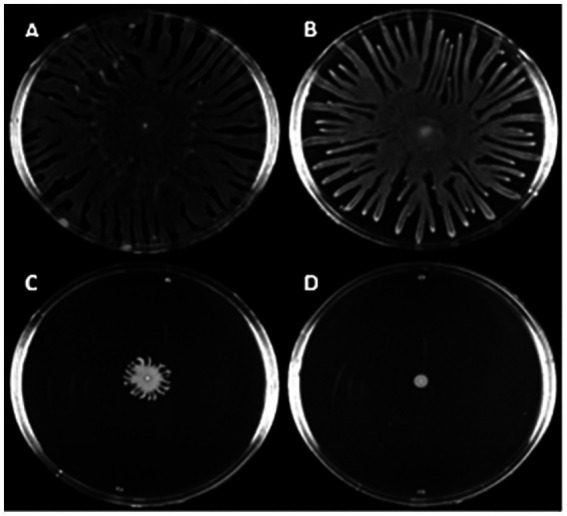
Canine OE isolates exhibit a range of swarming motility levels. Overnight cultures grown in LB were spotted onto swarming motility agar and incubated at 37° C for 16 h before imaging. Above shows selection of *P. aeruginosa* clinical canine otitis externa isolates with varying levels of swarming motility. **(A–C)** Shows strains PAO1, 83240 and C3524. **(D)** Shows strain 29775 which is swarming negative under the tested conditions. Images shown are representative of three biological repeats.

Most strains (71%, 179/253) tested positive for swarming motility (Figure 6). The positive control strain (PAO1) consistently migrated to the perimeter of the agar plate within 16 h of incubation, and 16% (41/253) of the clinical isolates showed a similar swarming ability. Of the 205 isolates that produced strong levels of biofilm, 72% (147) also tested positive for swarming. Interestingly, strain 29051—the strongest biofilm producer—tested negative for swarming. When the relationship between biofilm formation and swarming motility was investigated no correlation was identified (r^2^ = 0.002; Supplementary Figure 4).

### Presence of virulence genes in clinical isolates

**Figure 7:**
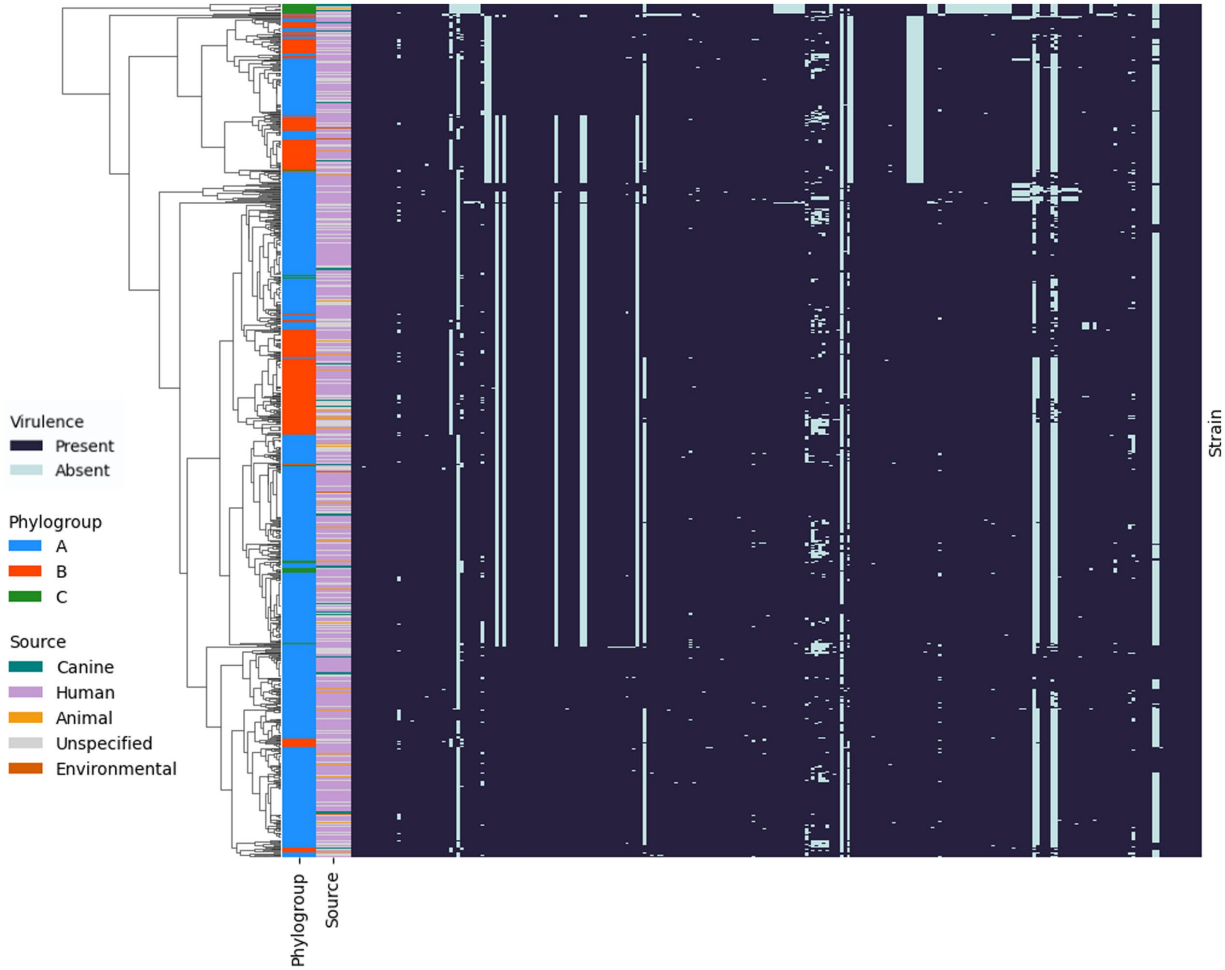
Presence and absence of virulence genes in the genomes of *P. aeruginosa* isolates. Hierarchical clustering using Jaccard distance and average linkage is shown on the left. Phylogroups A (blue), B (orange) and C (green) for each strain are shown in the outer band, with the source of the strains indicated in the inner band.

The presence and absence of virulence genes was investigated using ABRicate with VFDB. Comparison of the canine OE isolates from this study to other *P. aeruginosa* isolates from human, animal and environmental sources using hierarchical clustering highlighted that there is no specific virulence profile associated with canine OE infection (Figure 7). Additionally, this highlighted that the strains did not cluster within phylogroups based on virulence factors. However, one notable exception is a cluster of 12 strains– including some from canines—which lacked numerous virulence factors, namely a series of genes involved in type III secretion systems. The majority (92%; 11/12), of these isolates belonged to phylogroup C, specifically, the section of the tree identified as group 5 by Freschi et al., 2019. These results are in line with the aforementioned study as they also identified that isolates in this group were missing 36 genes that encode for a Type III secretion system. The remaining isolate belonged to phylogroup A and was missing 32/36 of the type three secretion genes.

## Discussion

Canine otitis caused by *P. aeruginosa* is a very common infection seen in veterinary practices worldwide which is frequently resistant to antimicrobial treatment. To our knowledge, this is the first study to use a combination of whole genome sequencing, phenotypic screening and bioinformatic analysis to characterise a large collection of clinical OE isolates of *P. aeruginosa* from diverse geographical origins. This revealed extensive genomic diversity across strains, as well as high levels of resistance to antimicrobials—particularly fluoroquinolones which are often used in the treatment of canine otitis. Furthermore, the MLST sequence type of two isolates (ST111, ST244) were identical to STs known to be associated with AMR nosocomial infections in people (Oliver et al., 2024), which may pose a risk of zoonotic spread, particularly to immunocompromised individuals. Interestingly, ST111 has been shown to have a low affinity to cystic fibrosis cases while ST244 has an intermediate affinity (Weimann et al., 2024).

MLST has been used previously to show that canine *P. aeruginosa* isolates are diverse (Hyun et al., 2018; Elfadadny et al., 2023). This is consistent with research on *Pseudomonas* infections in people, which also revealed a predominantly non-clonal population structure interspersed with highly successful epidemic clones and clonal complexes (Maatallah et al., 2011). These ‘high-risk’ clones include STs such as ST235, ST111, ST233 and ST244 (Oliver et al., 2024). Transmission of *Pseudomonas* between dogs and people has been reported in both directions (Fernandes et al., 2018; Santaniello et al., 2020). The identification of high-risk clones in dogs is particularly concerning considering the level of AMR detected, many of which are important in human medicine. Should this occur, the associated risks may increase over time given the ageing population in many countries, and higher dog ownership levels following the Covid-19 pandemic.

Antimicrobial resistance of isolates in this study was highest for fluoroquinolones. The levels of resistance reported here are similar to previous studies (Arais et al., 2016; Petrov et al., 2019; KuKanich et al., 2022). Fluoroquinolones are defined by the WHO as Critically Important Antimicrobials for human medicine, to be used only when alternative antimicrobials were ineffective (EMA, 2019). All strains resistant to ciprofloxacin were also resistant to levofloxacin and enrofloxacin suggesting that it is the most effective fluoroquinolone against *Pseudomonas in vitro*. This is most likely due to its lower affinity to efflux pumps compared to other fluoroquinolones (Teresa Tejedor et al., 2003). This is supported by strain 26491, which was resistant to enrofloxacin and levofloxacin but not ciprofloxacin and contained a mutation that is known to result in the overexpression of the MexXY-OprM efflux pump. Similarly, continuous exposure studies using *E. coli* from canine infections shows that QRDR mutations occur after treatment with marbofloxacin that can provide protection against other fluoroquinolones (Gebru et al., 2011). This is important because marbofloxacin is a component of antimicrobial containing otic treatments used in the United Kingdom.

Antimicrobial resistance gene (ARG) profiles amongst the isolates, as determined using AMRFinderPlus, were very similar. However, two integron associated genes, *aadA7* and *sul1*, were identified. Although these genes did not account for resistance to the antimicrobials tested in the present study, it does highlight that *P. aeruginosa* isolates in dogs can carry mobile genetic elements carrying antimicrobial resistance genes. This led to the investigation of mutations which identified five point mutations in the QRDR region that had previously been linked to a resistant phenotype. The presence of QRDR mutations has been studied in *P. aeruginosa* isolates from dog infections in Korea and Brazil where, similar to the present study, GyrA T83I was identified as a prevalent mutation (Arais et al., 2016; Park et al., 2020). This suggests that the high levels of fluoroquinolone resistance seen in the present study is likely due to QRDR mutations. Ultimately, this suggests that the use of antimicrobials in veterinary medicine can select for the development of mutations to clinically important human antimicrobials.

QRDR mutations do not account for all of the resistance seen in this study. *Pseudomonas aeruginosa* 84269 and 26491 were resistant to enrofloxacin/enrofloxacin and levofloxacin, respectively, but did not contain any mutations in the genes investigated. However, other well-characterised fluoroquinolone resistance mechanisms such as efflux pump overexpression and decreased outer membrane permeability could be responsible for the resistance observed as a mutation that introduced a premature stop codon into *mexZ*, which is known to result in efflux pump overexpression, was identified in 26491, suggesting that increased efflux might be the cause of this resistance (Hooper and Jacoby, 2016; López-Causapé et al., 2018). Moreover, 88812, which also had a *mexZ* mutation, was resistant to all the tested fluoroquinolones, in addition to amikacin and gentamicin. Although this isolate did have mutations in the QRDR, no resistance mechanisms for aminoglycosides were identified, again highlighting efflux as a potential resistance mechanism. However, it is thought that up to 200 genes could be involved in resistance to ciprofloxacin so it is possible that there are unknown mechanisms contributing to fluoroquinolone resistance (Bruchmann et al., 2013).

Worryingly, resistance to two carbapenem antimicrobials, meropenem and imipenem, was identified, with the latter present in 8.33% of isolates. This is significant as the World Health Organisation (WHO) recognises a critical need for new antimicrobials for the treatment of carbapenem resistant *P. aeruginosa* (WHO, 2017). Of the sequenced isolates in the present study, only one showed resistance to imipenem although no carbapenemase genes were identified. Carbapenemases have been found previously in a *P. aeruginosa* isolate from a case of canine otitis externa in Korea (Hyun et al., 2018).

Biofilm formation has been found in 40–100% of clinical *P. aeruginosa* from canine OE (Pye et al., 2013; Chan et al., 2019; Robinson et al., 2019). In our study, 96% of strains produced biofilm *in vitro*, with 82% classified as strong biofilm formers. Of the 4% of strains producing no detectable biofilm, only one (464429) was missing any of the biofilm genes that were screened for (specifically *pslABCD*).

Deletion of *pslD* can eliminate the ability of *P. aeruginosa* PAO1 to attach to a microtiter dish (Colvin et al., 2012). Some *P. aeruginosa* isolates have been shown to be deficient in *psl* production; most notably PA14, which is also missing *pslABCD* (Friedman and Kolter, 2004). As a result, PA14 uses *pel* as its primary biofilm matrix polysaccharide. Despite this, *P. aeruginosa* PA14 is still able to adhere to a microtiter dish and form a biofilm after 24 h (Colvin et al., 2012). This could mean that the strains that produce no quantifiable biofilm in the present study have an attachment deficiency. The presence of non-biofilm producing clinical strains is not yet fully understood, particularly given most of these have a full complement of biofilm-related genes. This might be explained by divergent expression in different environments, or deficiencies in transcriptional regulators such as LasR (Lima et al., 2018; Kamali et al., 2020).

Little is known about virulence factors specific to the development of canine OE. Investigation of five virulence genes in *P. aeruginosa* from canine sources, including the ear canal, found that three genes, *lasB*, *aprA*, and *plcH*, were present in all of the tested isolates, while *exoS* and *toxA* were present in 87.5 and 91.7%, respectively (Hattab et al., 2021). Similarly, in the present study *lasB*, *aprA*, and *plcH* were identified in all of the isolates and *toxA* in 91%.

*Pseudomonas aeruginosa* isolates usually harbour either *exoU* or *exoS*, although the reason for this is unclear. The distinction is important because *exoU^+^* strains are associated with more severe infections, and chronic OM infections in people (Park et al., 2017; Ozer et al., 2019). In the present study, *exoS^+^* strains constituted 69% (24/35) of the population with 14% (5/35) *exoU^+^*. Interesting, one isolate 3% (1/35) carried both genes and 14% (5/35) had neither. All the *exoU^+^* isolates in addition to the isolate containing both genes were located on the same branch of the phylogenetic tree as PA14 as previously described (Ozer et al., 2019). Although, other groups have reported isolates with both genes in phylogroup A (Botelho et al., 2023). None of the virulence genes identified in the canine isolates could be implicated in specifically having a role in this disease, as similar genes were also identified in environmental and human disease isolates.

In conclusion, our study has applied genome sequencing, phenotypic and bioinformatic analysis to a large collection of *P. aeruginosa* isolates from canine otitis externa. Although two of the isolates shared an MLST sequence type with high-risk STs from nosocomial human infections, there was little overlap of STs with previous studies. This genetic diversity was further supported by phylogenetic analysis which revealed no specific clustering of canine isolates; supporting the hypothesis that infections are acquired from environmental sources. Antimicrobial resistance was common, particularly towards the fluoroquinolones, which has implications for veterinary and human therapeutic failure. Although strains in this study did harbour known virulence and biofilm-associated genes, none of these were specifically associated with canine OE. These findings may help stimulate further research into the fundamental mechanisms of canine OE, and the optimising of therapy.

## Data Availability

The datasets presented in this study can be found in online repositories. The names of the repository/repositories and accession number(s) can be found in the article/Supplementary material.
