# Supplementary material for: Genomic and phenotypic characterisation of Pseudomonas aeruginosa isolates from canine otitis externa reveals high-risk sequence types identical to those found in human nosocomial infections
Source: Front Microbiol. 2025 Feb 24;16:1526843. doi: 10.3389/fmicb.2025.1526843 (PMC11891389; doi:10.3389/fmicb.2025.1526843)
Supplement: Supplementary file 1 [file Data_Sheet_1.docx]

| **Antibiotic** | **Concentration (μg)** |
| --- | --- |
| **Penicillin** | |
| Piperacillin | 100 |
| Piperacillin + Tazibactam | 100 + 10 |
| Ticarcillin + Clavulanic acid | 75 + 10 |
| **Cephalosporin** | |
| Ceftazidime | 30 |
| Cefepime | 30 |
| **Carbapenem** | |
| Imipenem | 10 |
| Meropenem | 10 |
| **Monobactam** | |
| Aztreonam | 30 |
| **Aminoglycosides** | |
| Amikacin | 30 |
| Gentamicin | 10 |
| Tobramycin | 10 |
| **Fluroquinolone** | |
| Enrofloxacin | 5 |
| Levofloxacin | 5 |
| Ciprofloxacin | 5 |

Supplementary Table 1 List of antibiotics used in the disc diffusion assay

| **Gene** | **Locus Tag** | **Description** |
| --- | --- | --- |
| *pelA* | PA3064 | hypothetical protein |
| *pelB* | PA3063 | pellicle/biofilm biosynthesis protein |
| *pelC* | PA3062 | pellicle/biofilm biosynthesis outer membrane protein |
| *pelD* | PA3061 | pellicle/biofilm biosynthesis protein |
| *pelE* | PA3060 | pellicle/biofilm biosynthesis protein |
| *pelF* | PA3059 | pellicle/biofilm biosynthesis glycosyltransferase |
| *pelG* | PA3058 | pellicle/biofilm biosynthesis Wzx-like polysaccharide transporter |
| *pslA* | PA2231 | biofilm formation protein PslA |
| *pslB* | PA2232 | biofilm formation protein PslB |
| *pslC* | PA2233 | biofilm formation protein PslC |
| *pslD* | PA2234 | biofilm formation protein PslD |
| *pslE* | PA2235 | biofilm formation protein PslE |
| *pslF* | PA2236 | biofilm formation protein PslF |
| *pslG* | PA2237 | biofilm formation protein PslG |
| *pslH* | PA2238 | biofilm formation protein PslH |
| *pslI* | PA2239 | biofilm formation protein PslI |
| *pslJ* | PA2240 | biofilm formation protein PslJ |
| *pslK* | PA2241 | biofilm formation protein PlsK |
| *pslL* | PA2242 | biofilm formation protein PslL |
| *pslM* | PA2243 | bFAD-binding dehydrogenase |
| *pslN* | PA2244 | hypothetical protein |
| *algD* | PA3540 | GDP-mannose 6-dehydrogenase AlgD |
| *alg8* | PA3541 | glycosyltransferase alg8 |
| *alg44* | PA3542 | alginate biosynthesis protein Alg44 |
| *algK* | PA3543 | alginate biosynthesis protein AlgK |
| *algE* | PA3544 | alginate production protein AlgE |
| *algG* | PA3545 | alginate-c5-mannuronan-epimerase AlgG |
| *algX* | PA3546 | alginate biosynthesis protein AlgX |
| *algL* | PA3547 | alginate lyase |
| *algI* | PA3548 | alginate o-acetylase AlgI |
| *algJ* | PA3549 | alginate o-acetylase AlgJ |
| *algF* | PA3550 | alginate o-acetyltransferase AlgF |
| *algA* | PA3551 | bifunctional mannose-1-phosphate guanylyltransferase/mannose-6-phosphate isomerase |
| *mvfR* | PA1003 | transcriptional regulator MvfR |
| *pqsA* | PA0996 | anthranilate--CoA ligase |
| *pqsB* | PA0997 | hypothetical protein |
| *pqsC* | PA0998 | hypothetical protein |
| *pqsD* | PA0999 | 3-oxoacyl-ACP synthase |
| *pqsE* | PA1000 | thioesterase PqsE |
| *algU* | PA0762 | RNA polymerase sigma factor AlgU |
| *mucA* | PA0763 | sigma factor AlgU negative regulator MucA |
| *mucB* | PA0764 | sigma factor AlgU regulator MucB |
| *mucC* | PA0765 | positive regulator for alginate biosynthesis MucC |
| *mucD* | PA0765 | serine protease MucD |
| *mucP* | PA3649 | metalloprotease protease |
| *mucE* | PA4033 | small envelope protein MucE |
| *algW* | PA4446 | AlgW protein |
| *algP* | PA5253 | alginate regulatory protein AlgP |
| *algQ* | PA5255 | anti-RNA polymerase sigma 70 factor |
| *algR* | PA5261 | alginate biosynthesis regulatory protein AlgR |
| *algZ* | PA5262 | alginate biosynthesis protein AlgZ/FimS |
| *algC* | PA5322 | phosphomannomutase |
| *algB* | PA5483 | two-component response regulator AlgB |

Supplementary Table 2 Biofilm associated genes investigated as part of the present study.

| Strain | gyrA_T83I | gyrA_D87H | gyrB_S466F | parE_A473V | parC_S87L | fusA1_Q678L | fusA1_T671A | oprD_V359L | mexZ_Q10STOP | mexZ_Q69STOP | pmrB_V15I |
| --- | --- | --- | --- | --- | --- | --- | --- | --- | --- | --- | --- |
| 2943 | 0 | 0 | 1 | 0 | 0 | 0 | 0 | 1 | 0 | 0 | 0 |
| 25181 | 0 | 0 | 0 | 0 | 0 | 0 | 0 | 0 | 0 | 0 | 1 |
| 26491 | 0 | 0 | 0 | 0 | 0 | 0 | 0 | 1 | 1 | 0 | 0 |
| 29582 | 0 | 0 | 0 | 0 | 0 | 0 | 0 | 1 | 0 | 0 | 0 |
| 29758 | 0 | 0 | 1 | 0 | 0 | 0 | 0 | 1 | 0 | 0 | 0 |
| 80632 | 0 | 0 | 0 | 0 | 0 | 0 | 0 | 1 | 0 | 0 | 0 |
| 80664 | 1 | 0 | 0 | 0 | 0 | 0 | 0 | 0 | 0 | 0 | 0 |
| 80715 | 0 | 0 | 0 | 0 | 0 | 0 | 0 | 0 | 0 | 0 | 1 |
| 83240 | 0 | 0 | 0 | 0 | 0 | 0 | 0 | 0 | 0 | 0 | 0 |
| 84269 | 0 | 0 | 0 | 0 | 0 | 0 | 0 | 1 | 0 | 0 | 0 |
| 85505 | 0 | 0 | 0 | 0 | 0 | 0 | 0 | 0 | 0 | 0 | 0 |
| 87895 | 1 | 0 | 0 | 0 | 1 | 1 | 0 | 0 | 0 | 0 | 0 |
| 88693 | 0 | 0 | 0 | 0 | 0 | 0 | 0 | 1 | 0 | 0 | 0 |
| 88812 | 1 | 1 | 0 | 0 | 1 | 0 | 0 | 1 | 0 | 1 | 0 |
| 26820-3 | 0 | 0 | 0 | 0 | 0 | 0 | 0 | 1 | 0 | 0 | 0 |
| 27827-1 | 0 | 0 | 0 | 0 | 0 | 0 | 0 | 0 | 0 | 0 | 1 |
| 29878-1 | 0 | 0 | 1 | 0 | 0 | 0 | 0 | 1 | 0 | 0 | 0 |
| 29878-2 | 0 | 0 | 1 | 0 | 0 | 0 | 0 | 1 | 0 | 0 | 0 |
| 463027 | 0 | 0 | 0 | 0 | 0 | 0 | 0 | 0 | 0 | 0 | 0 |
| 464429 | 0 | 0 | 0 | 0 | 0 | 0 | 0 | 0 | 0 | 0 | 1 |
| 467523 | 0 | 0 | 0 | 0 | 0 | 0 | 0 | 0 | 0 | 0 | 0 |
| 467896 | 0 | 0 | 0 | 0 | 0 | 0 | 0 | 1 | 0 | 0 | 0 |
| 480630 | 0 | 0 | 0 | 0 | 0 | 0 | 0 | 1 | 0 | 0 | 0 |
| 484098 | 0 | 0 | 0 | 0 | 0 | 0 | 0 | 0 | 0 | 0 | 1 |
| 484919 | 1 | 0 | 0 | 0 | 0 | 0 | 0 | 1 | 0 | 0 | 0 |
| 485101 | 0 | 0 | 0 | 0 | 0 | 0 | 0 | 0 | 0 | 1 | 0 |
| 488402 | 0 | 0 | 0 | 0 | 0 | 0 | 0 | 0 | 0 | 0 | 0 |
| 488427 | 0 | 0 | 0 | 0 | 0 | 0 | 0 | 0 | 0 | 0 | 0 |
| 488613 | 0 | 0 | 0 | 1 | 0 | 0 | 0 | 1 | 0 | 0 | 0 |
| 488958 | 1 | 0 | 0 | 0 | 0 | 0 | 1 | 1 | 0 | 0 | 0 |
| 489267 | 1 | 0 | 1 | 0 | 1 | 0 | 0 | 1 | 0 | 0 | 0 |
| 490051 | 0 | 0 | 0 | 0 | 0 | 0 | 0 | 0 | 0 | 0 | 0 |
| 490137 | 0 | 0 | 0 | 1 | 0 | 0 | 0 | 1 | 0 | 0 | 0 |
| 490614 | 0 | 1 | 0 | 0 | 0 | 0 | 0 | 0 | 0 | 0 | 0 |
| C5752-1 | 0 | 0 | 0 | 0 | 0 | 0 | 0 | 0 | 0 | 0 | 0 |

Supplementary Table 3 Presence and absence of point mutations identified by AMRFinderPlus


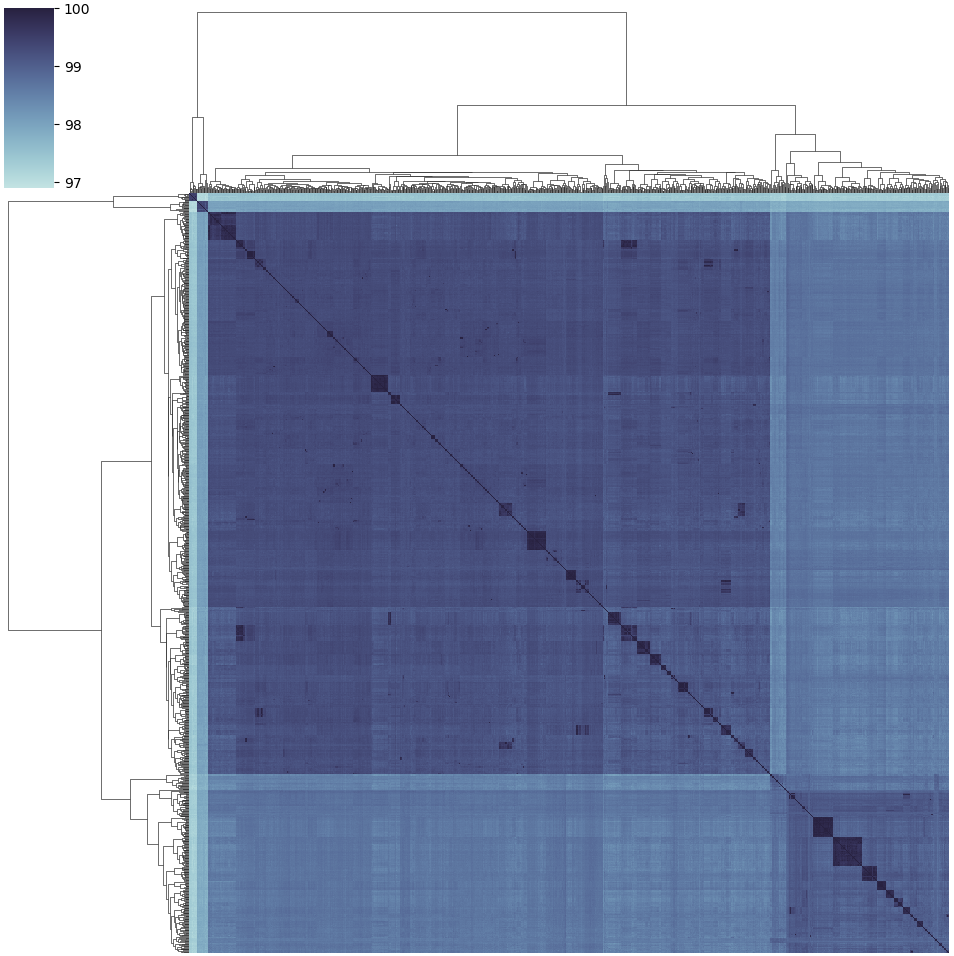


Supplementary Figure 1Hierrchical clustering with complete linkage of whole genome average nucleotide identity (ANI) between 1035 *P. aeruginosa* isolates calculated using FastANI.


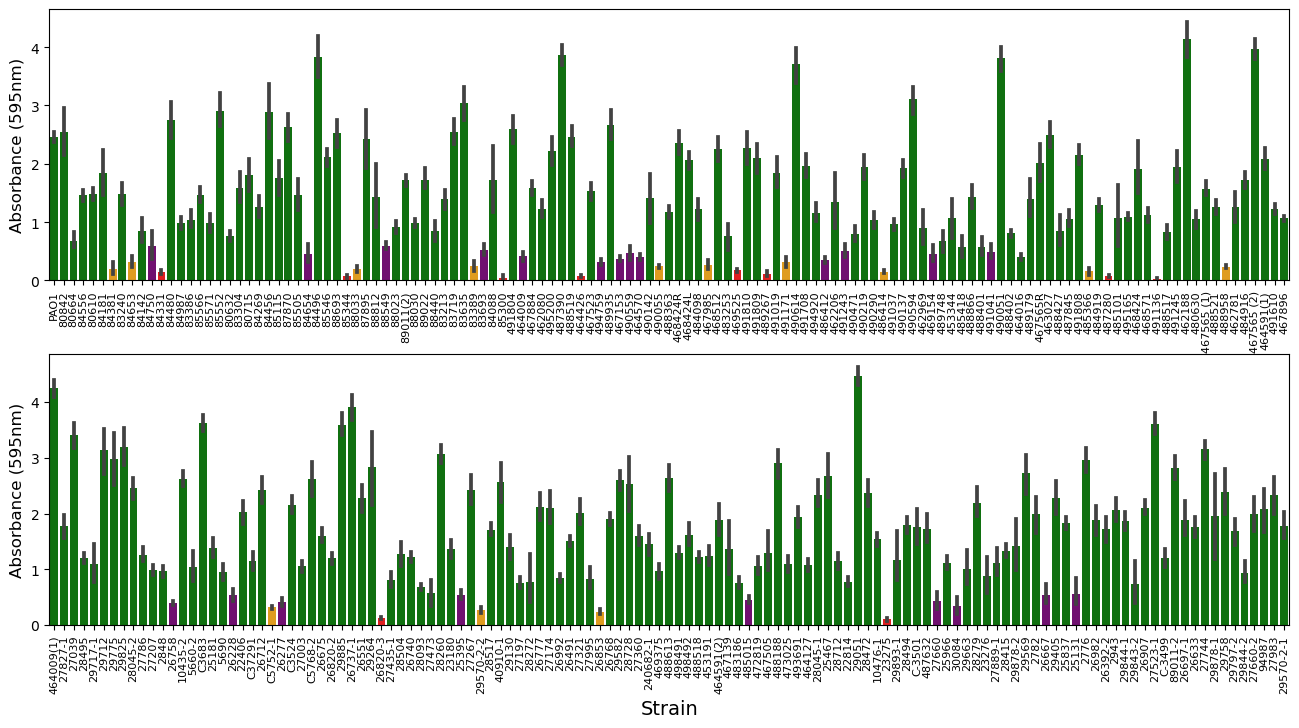


Supplementary Figure 2 Biofilm formation of *P. aeruginosa* from clinical cases of canine otitis externa. The biofilm forming ability of 253 *P. aeruginosa* strains was assessed using a 96 well plate, crystal violet assay. Green – Strong biofilm producing - Purple – Moderate biofilm producing - Amber – Weak biofilm producing - Red – No quantifiable biofilm. The values here are from three biological and six technical repeats represented by the mean with 95% confidence interval.


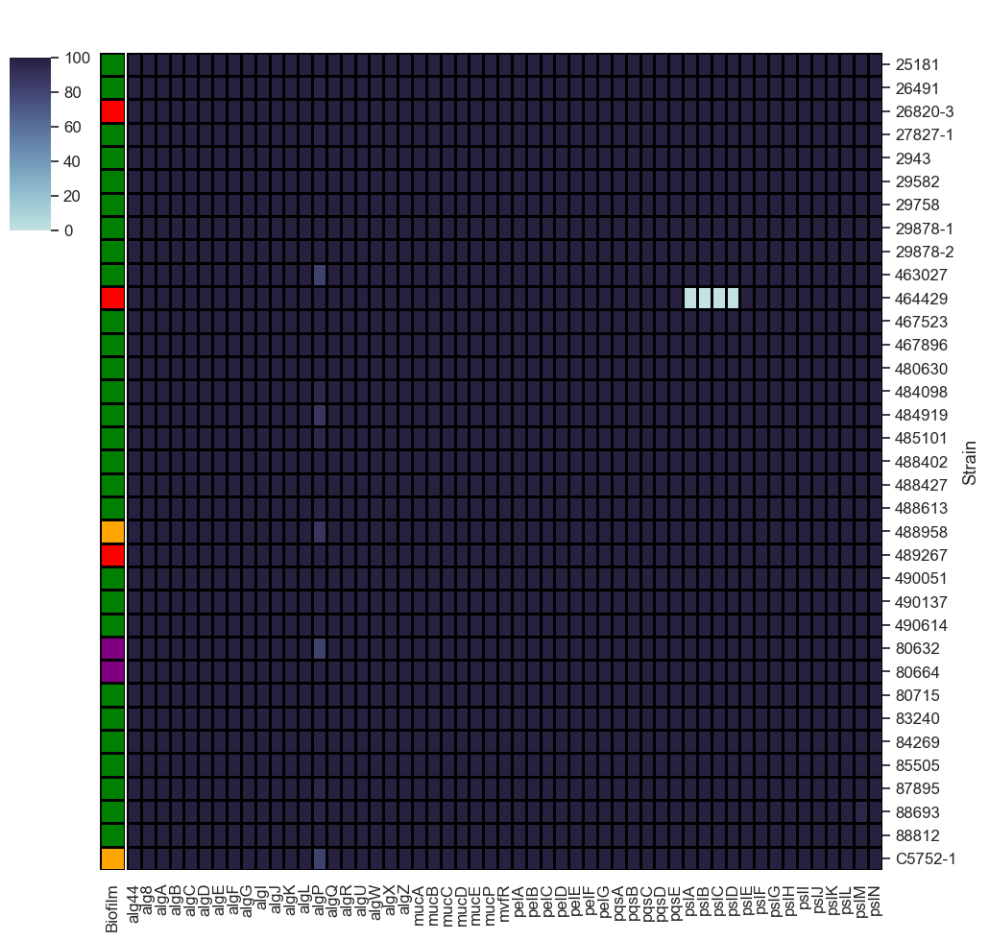


Supplementary Figure 3 Percentage coverage of the nucleotide sequence of 53 genes associated with biofilm formation in Pseudomonas aeruginosa. Heatmap displaying percentage coverage of the genomes of 35 P. aeruginosa canine OE isolates to 53 genes from PAO1 that are associated with biofilm formation. Results from a phenotypic assay of biofilm formation are displayed on the left bar: Green – Strong biofilm producing - Purple – Moderate biofilm producing - Amber – Weak biofilm producing - Red – No quantifiable biofilm


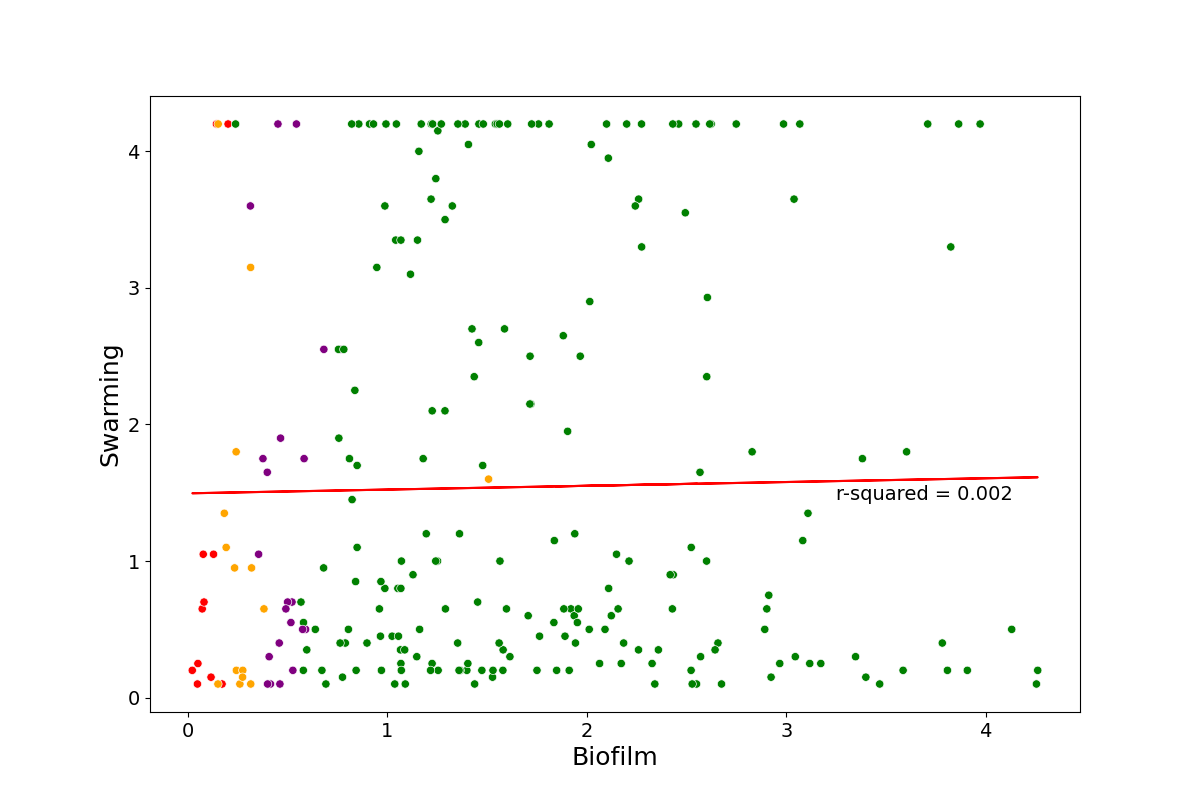


Supplementary Figure 4 A scatter plot showing the relationship between absorbance in a crystal violet biofilm assay and distance travelled in a swarming motility assay for 253 clinical *P. aeruginosa* isolates from canine otitis externa. Results from the biofilm assay are indicated on the graph - Green – Strong biofilm producing - Purple – Moderate biofilm producing - Amber – Weak biofilm producing - Red – No quantifiable biofilm. Swarming motility was measured in cm.
